# Individual and Interactive Effects of Socio-Ecological Factors on Dengue Fever at Fine Spatial Scale: A Geographical Detector-Based Analysis

**DOI:** 10.3390/ijerph14070795

**Published:** 2017-07-17

**Authors:** Zheng Cao, Tao Liu, Xing Li, Jin Wang, Hualiang Lin, Lingling Chen, Zhifeng Wu, Wenjun Ma

**Affiliations:** 1 Guangzhou Institute of Geochemistry, Chinese Academy of Sciences, Guangzhou 510640, China; jnczdl@163.com (Z.C.); egmontwj@gmail.com (J.W.); 2 University of Chinese Academy of Sciences, Beijing 100049, China; 3 Guangdong Provincial Institute of Public Health, Guangdong Provincial Center for Disease Control and Prevention, Guangzhou 511430, China; gztt_2002@163.com (T.L.); lixing.echo@foxmail.com (X.L.); Linhualiang2002@163.com (H.L.); 4 School of Geographical Sciencesof Guangzhou University, Guangzhou 510006, China; Lingling_Chen_00@163.com

**Keywords:** dengue fever, socio-ecological factors, geographical detectors, GIS and RS

## Abstract

*Background*: Large spatial heterogeneity was observed in the dengue fever outbreak in Guangzhou in 2014, however, the underlying reasons remain unknown. We examined whether socio-ecological factors affected the spatial distribution and their interactive effects. *Methods*: Moran’s I was applied to first examine the spatial cluster of dengue fever in Guangzhou. Nine socio-ecological factors were chosen to represent the urbanization level, economy, accessibility, environment, and the weather of the 167 townships/streets in Guangzhou, and then the geographical detector was applied to analyze the individual and interactive effects of these factors on the dengue outbreak. *Results*: Four clusters of dengue fever were identified in Guangzhou in 2014, including one hot spot in the central area of Guangzhou and three cold spots in the suburban districts. For individual effects, the temperature (*q* = 0.33) was the dominant factor of dengue fever, followed by precipitation (*q* = 0.24), road density (*q* = 0.24), and water body area (*q* = 0.23). For the interactive effects, the combination of high precipitation, high temperature, and high road density might result in increased dengue fever incidence. Moreover, urban villages might be the dengue fever hot spots. *Conclusions*: Our study suggests that some socio-ecological factors might either separately or jointly influence the spatial distribution of dengue fever in Guangzhou.

## 1. Introduction

Dengue fever (DF) is an infectious disease transmitted by *Aedes albopictus* and *Aedes aegypti* [1]. The World Health Organization (WHO) has estimated that more than 50 million dengue cases occur each year and almost half of the world’s population live in areas where dengue is endemic [2,3]. Along with the urbanization, both spatial and temporal expansions of dengue fever have been reported worldwide. Understanding the interaction between dengue fever and potential risk factors (geography, environment, and socioeconomic status) is important, which in turn is related to the spatial-temporal heterogeneity of dengue fever, and will help the government take appropriate control measures [4,5,6,7].

Previous studies have investigated the impacts of socio-ecological factors on dengue fever outbreaks and transmission. Hunter et al. examined the relationship between the construction of water impoundments and dengue fever in tropical areas, and found that the increasing water areas exacerbated the transmission of dengue fever, because they served as breeding places for mosquitos [8]. Other studies have also been conducted to further investigate these associations [9,10,11,12]. For example, Qi et al. [13] explored the impact of socio-ecological factors on dengue fever outbreak in China, and revealed that high road density and low gross domestic product (GDP) per capita were important risk factors of dengue fever, and high population density and normalized difference vegetation index (NDVI) could result in an increase in dengue fever cases. They suggested that the areas with low GDP and high population density were usually peri-urban areas with poor hygiene condition, which could promote the increase of vector cluster, and hence increase the risks of dengue infection. Hu examined the socio-ecological drivers of dengue fever transmission in Queensland using Bayesian approaches, and found that the incidence of dengue fever was highly associated with the monthly average temperature, monthly average rainfall, population density, and overseas travelers [14].

Though those previous studies showed that dengue fever was influenced by socio-ecological factors, several limitations are worth considering in future studies. First, majority of previous studies employed temporal methods, such as time-series study design to explore risk factors of dengue outbreaks [12,15], and only a few have considered the spatial heterogeneity of dengue case distribution [16,17]. Furthermore, the studies that considered the spatial heterogeneity of dengue cases were mostly conducted on a coarse spatial resolution (provincial or city) rather than on a fine scale [17,18,19]. Finally, almost all previous studies focused on the individual impacts of each socio-ecological factors on dengue fever [20,21], and few investigated the interaction of these factors. Filling these knowledge gaps are helpful for deepening the understanding of the transmission of dengue fever and allocating resources for prevention and control of dengue fever precisely.

To fill these knowledge gaps, we attempted to examine the individual and interactive impacts of socio-ecological factors on dengue fever at a fine scale using the geographical detector in this study, which is based on spatial variance analysis of the spatial consistency of health risk or disease distribution with suspected socio-ecological factors [22]. The findings of this study will be useful to identify the dominant driving factors of dengue fever, which is important for policy decision and resource allocation.

## 2. Materials and Methods

### 2.1. Study Site

**Figure 1:**
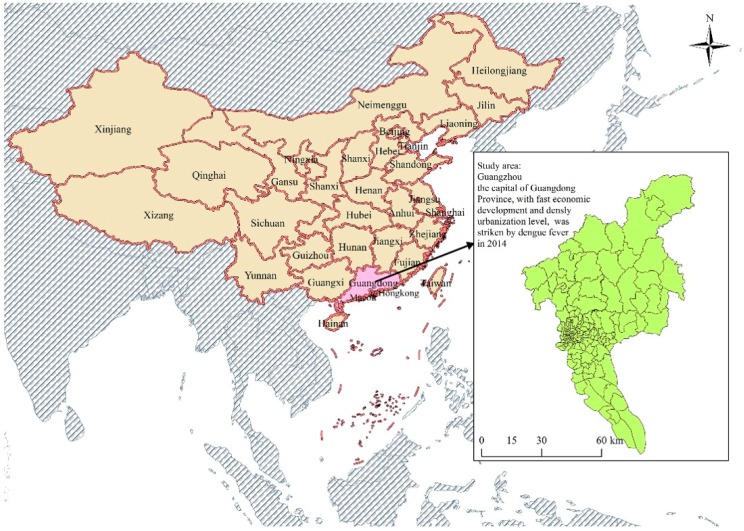
Geographical location of townships/street in the study area of Guangzhou, Guangdong, China.

Guangzhou, the biggest city and the economic, political, and cultural center of Guangdong Province, is located between the 22°26′ to 23°56′ N and 112°57′ to 114°3′ E (Figure 1). A total of 167 townships/streets, which are the smallest government administrative units comprised of several communities, were included in the study area. The population in 2010 is more than 12 million (according to the 2010 National Population Census). Guangzhou is one of the areas severely affected by dengue fever outbreaks [23,24]. In the last three decades, the incidence of dengue fever has increased by nearly 300%, from 497 in 1987 to 1265 in 2013. In particular, an explosive outbreak occurred in 2014, resulting to a total of 37,322 cases and five deaths [25].

### 2.2. Data

#### 2.2.1. Dengue Fever Data

Daily data of dengue fever in Guangzhou in 2014 were obtained from the National Notifiable Infectious Disease Reporting Information System, which has access to the Guangdong Provincial Center for Disease Control and Prevention [26]. Only clinically or laboratory confirmed indigenous dengue fever cases are included in this study. According to the diagnostic criteria for dengue fever enacted by the Chinese Ministry of Health, a patient was considered as a confirmed case if the dengue fever virus RNA was detected in their serum using real-time PCR or if IgM against dengue fever virus was present.

A patient was considered to be a clinically diagnosed case if he or she had an acute onset of rashes, headache, subjective fever, itching, anorexia, and leukopenia, and lived in the dengue fever outbreak area [27]. All data were anonymous without individual identifiable information. The surveillance was determined by the Chinese Ministry of Health as part of continuing public health surveillance and was exempted from institutional review board assessment [28]. The dengue fever case data was classified by townships/street based on the home addresses. During the outbreak in 2014, the first confirmed case was observed in 1 June, the epidemic peak with 1596 dengue cases was observed in 1 October, and the last case was observed in 12 December.

#### 2.2.2. Socio-Ecological Data

##### Urbanization Data

Three factors were chosen to describe different aspects of urbanization at townships/street level, including population density, urbanization level, and the ratio of urban village. Because the population density data in Guangzhou were not available in 2014, we used the 2015 data as a replacement. The population density data (persons/km^2^) of each township/street in Guangzhou was downloaded from the Socioeconomic Data and Applications Center hosted by the Columbia University (http://beta.sedac.ciesin.columbia.edu/) [16,29]. Urbanization level was defined as the ratio of construction land in each township/street. Construction land data of 2014 was obtained from the Engineering Technology and Research Center of Guangdong Geography Census Monitoring and Comprehensive Analysis based on Landsat 8 satellite data. The ratio of urban village in 2014 stands for the high-density and low-rise building areas, which was estimated using the remote sensing (RS) technology. First, the impervious surface was derived from the Landsat8-OLI (15 October 2014, Greenwich Mean Time) and Quickbird (5 October 2014, Greenwich Mean Time) satellite imagery, and then a threshold value was set to select the highest density area as the urban village areas [30]. An urban village in China was developed during the rapid urbanization process. Many farmlands were transformed into construction land, which formed the urban fringe areas. Migrants came into the urban city and lived in the urban fringe areas, which were gradually surrounded by the highly urbanized area and turned into an urban village [31]. Guangzhou has more than 100 urban villages, which were distributed in nearly every district of Guangzhou. Geography census data and urban village results were obtained from the Engineering Technology and Research Center of Guangdong Geography Census Monitoring and Comprehensive Analysis.

##### Economy

Data on Gross Domestic Product (GDP) per capita in 2010 at the township/street level in Guangzhou were retrieved from the National Bureau of Statistics of China, which was computed by multiplying the population and GDP per capita at the township/street level. We used the data in 2010 because this is the only available data closest to 2014. Road density was selected to reflect the accessibility of Guangzhou, and the road net data were obtained from website of the GEOFBRIK (http://www.geofabrik.de/index.html). We classified roads into eight types based on their location, administration, and utility, i.e., footway, living street, path, pedestrian, residential, road, step, and unclassified. All eight types in 2014 were used to calculate the road density.

##### Environmental Factors

Normalized difference vegetation index (*NDVI*) was acquired by the Moderate Resolution Imaging Spectroradiometer (MODIS) satellite imagery to represent the greenness of vegetation and photosynthetic rate. NDVI is an indispensable factor to assess whether the target areas contain living vegetation. The monthly NDVI data (MOD13A3) was obtained, and the annual NDVI value in 2014 was calculated. As NDVI can only reflect the greenness of target areas but not the vegetation coverage ratio, we applied NDVI to calculate the vegetation fraction (VFC) using the following equation [32]:

(1) $$VFC=\frac{NDVI-NDVI_{min}}{NDVI_{max}-NDVI_{min}}\times 100\%$$

where *NDVI* denotes the pixel *NDVI* value, *NDVI_min_* denotes the bare soil *NDVI* value, and *NDVI_max_* denotes the live vegetation *NDVI* value. The vegetation fraction (VFC) was calculated using the ENVI5.0 software (version 5.0, ESRI, Redlands, CA, USA). The resolution of VFC was 1 km, but the urbanization level and ratio of urban village were 30 m, and all the satellite-derived data were unified into 1 km × 1 km resolution, although this may lose some information of the precise profile of the urban village area. Water body areas were chosen to reflect the environmental aspects. Water body data in 2014 in Guangzhou were obtained from the Engineering Technology and Research Center of Guangdong Geography Census Monitoring and Comprehensive Analysis.

##### Meteorological Data

According to previous investigations [15], temperature and precipitation were chosen to represent climatic conditions. Meteorological data were obtained from the Guangdong Meteorological Service, including daily mean temperature and total rainfall of 127 automatic meteorological stations in Guangzhou. According to the literature, meteorological factors have zero- to three-month lag effect on dengue fever [33]. Therefore, temperature and precipitation from 1 May to 31 October were selected. Temperature and rainfall at township/street level were obtained using Kriging interpolation through ArcGIS (version 10.2, ESRI, Redlands, CA, USA). Then, the Advanced Spaceborne Thermal Emission and Reflection Radiometer Global Digital Elevation Model (ASTER GDEM) data (http://www.gscloud.cn/) were applied to calibrate the temperature with a lapse rate of 6 °C/km. For precipitation, a Kriging interpolation method was used first to obtain the precipitation interpolation result. Then, the multivariate regression relationship of *Y*_precipitation_ = *X* (latitude, longitude, elevation) was established. Finally, the interpolated precipitation result was adjusted based on the multivariate regression relationship [34].

### 2.3. Method

#### 2.3.1. Moran’s I

Moran’s I measures the spatial autocorrelation using the following equation [35]:

(2) $$I_{i}=\frac{x_{i}-\bar{x}}{s^{2}}\underset{j}{\sum }c_{ij}\left(x_{j}-\bar{x}\right)$$

where $x_{i}$ is the attribute value of spatial grid i, c is the matrix of spatial weights, $c_{ij}$ represents the weights of relationship between grid i and j, and $\bar{x}$ and $s^{2}$ represent the mean value and variance of x, respectively. $I_{i}$ is divided into two parts: positive and negative values. $I_{i}$ has a positive value when the grid i is similar to its neighbors, whereas it is negative when the grid i is dissimilar to its neighbors. The Moran’s I detected clusters and were classified into five categories: high-high, low-low, high-low, low-high, and non-significant. The high-high areas indicated the hot spots of dengue fever incidence, while the low-low areas implied the cold spots of dengue fever incidences. High-low and low-high areas were the outliers.

#### 2.3.2. Geographical Detector

Geographical detector was used to assess the impact of environmental factors on disease occurrence or transmission by evaluating the spatial heterogeneity between environmental factors and the diseases. The hypothesis was that the environmental factors would have the similar spatial distribution with the disease, if the chosen environmental factors contribute to the disease occurrence or transmission. This method has been applied in the environmental-related disease investigation, such as typhoid and paratyphoid fever [36]; hand, foot, and mouth diseases [37]; and neural tube defects [38]. The assessment was measured using the following equation:

(3) $$q=1-\frac{1}{N\sigma^{2}}\overset{L}{\underset{h=1}{\sum }}\left(N_{h}\sigma_{h}^{2}\right)$$

where *N* and $\sigma^{2}$ refer to the area and the dispersion variance of disease incidence, respectively. The study area is stratified into *L* stratums, *h* = 1, 2, 3 … *L*. In this study, *L* is the number of townships/street in Guangzhou. *q* was defined as the spatial heterogeneity attribute whose statistical properties change in space. The range of *q* is from 0 to 1, where 1 implies the disease is completely controlled by the chosen environmental factor, while 0 means the disease is totally unrelated to the chosen environmental factor.

Interactive effect was applied to determine whether factors *X*_1_ and *X*_2_ weaken or enhance one another when they work together. Layers *X*_1_ and *X*_2_ were overlaid and their attributes were combined (*X*_1_∩*X*_2_) as a new attribute, *X*_3_. The explanation levels of *X*_1_, *X*_2_, and *X*_3_ were calculated using the geographical detector, respectively. The interactive effects were judged using the following rules with explanation levels of *X*_1_, *X*_2_, and *X*_3_:

Enhance, nonlinear: *q*(*X*_1_∩*X*_2_ = *X*_3_) > *q*(*X*_1_) + *q*(*X*_2_) (4)

Independent: *q*(*X*_1_*∩X*_2_ = *X*_3_) = *q*(*X*_1_) + *q*(*X*_2_) (5)

Enhance, bi: *q*(*X*_1_*∩X*_2_ = *X*_3_) > Max (*q*(*X*_1_), *q*(*X*_2_)) (6)

Weaken, uni-: Min (*q*(*X*_1_), *q*(*X*_2_)) < *q*(*X*_1_*∩X*_2_ = *X*_3_) < Max (*q*(*X*_1_), *q*(*X*_2_)) (7)

Weaken, nonlinear: *q*(*X*_1_*∩X*_2_ = *X*_3_) < Min (*q*(*X*_1_), *q*(*X*_2_)) (8)

where *q*(*X*_1_), *q*(*X*_2_), *q*(*X*_1_∩*X*_2_ = *X*_3_) refers to explanation levels of *X*_1_, *X*_2_ and *X*_3_ conducted using the geographical detector. Min (*q*(*X*_1_), *q*(*X*_2_)), Max (*q*(*X*_1_), *q*(*X*_2_)) refers to the minimum and maximum values of *X*_1_ and *X*_2_ explanation levels.

Moran’s I was applied to detect the spatial cluster of dengue fever, while the geographical detector was used to examine the individual and interactive impact of socio-ecological factors on dengue fever.

## 3. Results

### 3.1. General Information of the Dengue Epidemic

In 2014, 37,322 indigenous dengue fever cases were reported in Guangzhou. The average incidence rate was 289.97 per 100,000 population. Women accounted for 50.73% with an average incidence rate of 307.39 per 100,000 population. Elderly individuals had the highest incidence rate (497.64 per 100,000 population). Among the occupations, household/unemployed represented the largest proportion (23.36%) of indigenous dengue fever cases, which is followed by the retired (13.99%). The general information of dengue fever in Guangzhou in 2014 is shown in the Supplementary Table S1.

**Figure 2:**
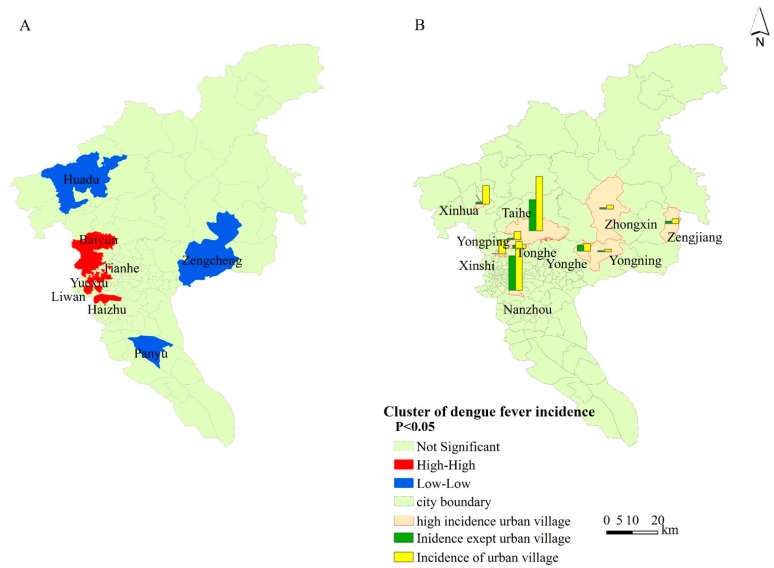
Dengue fever spatial cluster at the district level and the high dengue fever incidence across townships/streets in Guangzhou in 2014. (**A**) is the spatial cluster of dengue fever, and (**B**) is the comparison of urban villages with non-urban villages in the same township/street in the top ten dengue incidence townships/streets.

In Figure 2A, one hot spot (high incidence area) was detected in the center area of Guangzhou, including, five districts (Baiyun, Tianhe, Yuexiu, Liwan, and Haizhu), while three cold spots (low incidence areas) were found in the outskirts (Huadu, Panyu, and Zengcheng). Moreover, at the township/street level, 10 high dengue fever incidence areas were identified (Figure 2B). Dengue fever incidence in urban villages was higher than that of non-urban villages in the same township/street. A total of four high dengue fever incidence were found in the urban villages in Baiyun district, one in Haizhu district, one in Huadu district, one in Huangpu district, and three in Zengcheng district.

### 3.2. Socio-Ecological Distribution at Township/Street Level in Guangzhou

**Figure 3:**
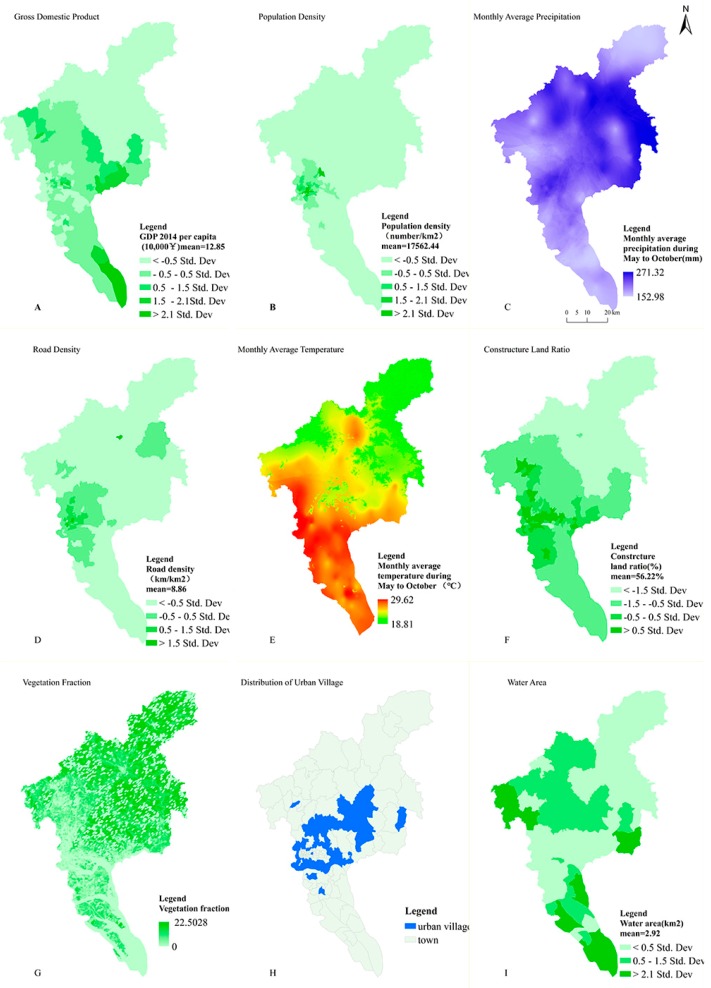
Spatial distribution of socio-ecological factors in 167 townships/streets in Guangzhou in 2014. (**A**) Gross domestic product (GDP) per capita; (**B**) Population density; (**C**) Monthly average precipitation from May to October; (**D**) Road density; (**E**) Monthly average temperature from May to October; (**F**) Construction land ratio; (**G**) Vegetation fraction; (**H**) Urban village; (**I**) Water areas.

Nine socio-ecological factors were mapped in Figure 3.

The mean values of these factors were 128,500 CNY (GDP per capita, A), 17,562.44 person per km^2^ (population density, B), 204.81 mm (precipitation, C), 8.86 km/km^2^ (road density, D), 27.43 °C (temperature, E), 56.22% (urbanization level or construction land ratio, F), 16.47% (VFC, G), 11.50 km^2^ (urban village, H), and 2.92 km^2^ (water areas, I). Most areas with high value of socio-ecological factors were located in the center or south of Guangzhou, while precipitation was heavy in the northeast of areas, and the majority of water bodies were located in northwest and south areas. 

### 3.3. Assessing the Impact of Socio-Ecological Factors on Dengue Fever

Table 1 showed that dengue fever incidence was positively associated with the population density (*R* = 0.49, *p* < 0.01), road density (*R* = 0.36, *p* < 0.01), temperature (*R* = 0.51, *p* < 0.01), urbanization level (*R* = 0.42, *p* < 0.01), the ratio of urban villages (*R* = 0.28, *p* < 0.01), and precipitation (*R* = 0.09, *p* < 0.01). However, GDP per capita (*R* = −0.23, *p* < 0.01), VFC (*R* = −0.57, *p* < 0.01), and water body areas (*R* = −0.38, *p* < 0.01) were negatively correlated with the incidence. The results of geographical detector showed that seven factors, including precipitation, road density, temperature, urbanization level, VFC, urban village, and water body areas, are statistically significant (*p* < 0.05), but the GDP per capita and population density were not significant. Monthly average temperature (*q* = 0.33) was the dominant factor of dengue fever, followed by monthly average precipitation (*q* = 0.24), road density (*q* = 0.24), and water body area (*q* = 0.23).

### 3.4. Interaction of Socio-Ecological Factors on Dengue Fever

When only the interactive impacts of two socio-ecological factors were taken into consideration, 32 pairs of joint impacts were statistically significant, apart for four pairs (GDP and urban village ratio, GDP and population density, urbanization level and VFC, and road density and VFC). All the interactive impact results are shown in Supplementary Table S2.

**Figure 4:**
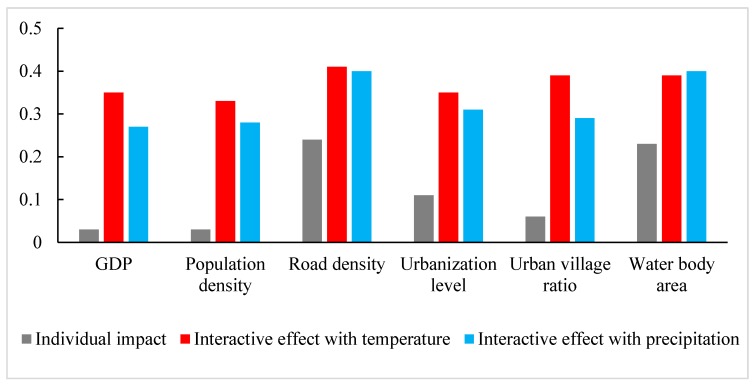
Interactive impact of weather factors and other socio-ecological factors. Gray is the individual impact of GDP, population density, road density, urbanization level, urban village ratio, and water body area. Red is the interactive impact of temperature and the above socio-ecological factors. Blue is the interactive impacts of precipitation and the above socio-ecological factors. All the interactive impacts are statistically significant (*p* < 0.05).

Figure 4 shows that when temperature and precipitation are taken into consideration, the interaction of other socio-ecological and meteorological factors increases dramatically. The combination of weather and other socio-ecological factors is nearly 1.5–12 times larger than the individual impact of GDP, population density, etc.

## 4. Discussion

Nowadays, dengue fever has been regarded as the most prevalent and rapidly spreading mosquito-borne viral disease in humans. Exploring the risk factors and implementing control interventions are the major strategies to control and prevent global dengue epidemics. In the present study, we used a spatial analysis method to investigate the independent and interactive effects of several socio-ecological factors on the 2014 dengue epidemic at a township/street level in Guangzhou. We observed four clusters of dengue cases here; while the dengue infection risks demonstrated positive associations with road density, ambient temperature, urbanization level, urban village ratios, and precipitation, but negative associations with GDP per capita, VFC, and water body areas.

The effects of population density, road density, urbanization, urban village, and GDP level on dengue epidemics have been assessed in several studies previously [13,17,39,40]. For example, Hassan et al. observed that high risk of dengue infections mainly appeared in the areas with higher population density and low neighborhood quality in Saudi Arabia [17]. Qi et al. also observed that the risks of dengue infection were higher in areas with higher population density, higher road density, and lower GDP per capita in the Pearl River Delta in China [13]. However, we did not find significant effects of population density on dengue incidence in this study. This inconsistency with some previous studies might be explained by several reasons. First, during the epidemic of dengue outbreak, more rigorous control interventions were conducted in the areas with high population density [41], such as in the central urban areas, which might more sufficiently control the transmission and outbreak of dengue. Second, the methods used in most previous studies did not take into account the spatial heterogeneity of dengue case distribution [15,42,43]. We here employed a geographical detector, which could sufficiently adjust for the spatial heterogeneity between the study units and may provide more precise results [22]. However, our study only focused on a single city, and it is needed to be further clarified in future studies.

Urban villages were found to have a higher dengue incidence rate compared with other areas, which was similar to that of Saudi et al.’s study [17]. Urban villages in China have poor quality living areas. It differs from that in the Western countries, which refers to a village-style urban neighborhood [44]. Urban villages in Guangzhou were built without unified planning, and the sanitation such as poor garbage transfer and disposal system was poor [31]. Water pits were easily formed in the villages, which could provide breeding sites for mosquitos. In addition, the distances between buildings are usually very small, which can cause ideal shadow places for mosquito abundance because *Aedes albopictus* tended to be the semi domestic mosquitoes and semi wild living mosquitoes, and it usually established breeding sites under shadowed water containers or near construction site pool [45,46]. In addition, more than 70% of the urban village residents have low educational attainment, which results in a lack of knowledge regarding dengue prevention [47,48], and leads to higher risk of being infected with dengue. These findings imply that improving dengue prevention and control strategies in urban village areas is quite urgent.

We further observed that environmental factors, including VFC and water body areas, were negatively related to dengue incidences, indicating that areas with more vegetation and water body had lower dengue incidence, which is consistent with several previous studies [12,13,49]. The negative associations may be because the areas with higher NDVI and more water body were mainly located in the suburbs of Guangzhou, where the mosquito density may be very high because their habitats are usually present in these areas, but that of the population density was very low. For example, in the Conghua district (a district located in the north of Guangzhou) in 2014, although the average Breteau Index (BI, an index used to evaluate the number of containers positive for *Aedes* larvae or pupae per 100 houses [50]) was higher than that in the Yuexiu district located in the central urban area (3.28 vs. 0.88), the average population density was 1231 person/km^2^, which was significantly lower than that in the Yuexiu district (45,393 person/km^2^). Therefore, even though some people are infected by dengue virus, the virus may be difficult to be transmitted to other people [51,52]. However, some other studies found positive associations between dengue epidemics and vegetation and surface water areas [42,53]. The reasons for these differences are still unknown. Therefore, more studies are needed to further investigate the effects of vegetation and water body on dengue fever risks in the future.

The positive associations between dengue incidence and temperature and precipitation in our study are similar to some previous studies [54,55,56,57,58]. For instance, Wu et al. who conducted a study in Taiwan, with the same latitude as Guangzhou, observed that dengue fever incidence was positively related with monthly maximum temperature, minimum temperature, and rainfall at different lag months [58]. Xu et al. also found similar results in Guangzhou from 2005 to 2015 using generalized additive models (GAMs) and zero-inflated GAMs (ZIGAMs) [57]. It has been demonstrated that ambient temperature affects each stage of the mosquito’s life cycle. High temperature could shorten the incubation period and increase the biting rates, which may enlarge the mosquito population in a short period of time. High temperature could also increase human exposure to mosquitos, e.g., by the increased time of spending outdoors and opening windows [59]. Precipitation is another important factor affecting dengue fever incidence mainly through the effects on mosquito’s life cycle and population dynamics. Increased near-surface humidity has been associated with precipitation, which could enhance mosquito flight activity and host-seeking behaviors, and rainfall can also change the abundance and type of aquatic habitats available to the mosquitos for egg depositions (oviposition) [60]. In addition, precipitation can provide breeding sites and promote egg hatching, which causes an increase in the number of mosquitoes [61]. Our observations illustrated that temperature and precipitation are important factors of dengue infection in China and could be used to predict or project dengue transmission. 

For interactive impacts, we mainly found that temperature and precipitation could significantly enhance other factors’ effects on dengue fever, which indicates that they may be the dominant determinants of dengue fever in South China, which is consistent with that of a previous study [62]. The average temperature in Guangzhou ranged between 18.81 °C and 29.62 °C. Therefore, increase in average temperature caused by urban heat island could significantly increase the risks of dengue fever infection in the central urban areas. The increase of precipitation in the central areas could increase breeding sites for mosquitos, leading to increased density of mosquitos, and hence increasing the transmission risks of dengue fever virus [63]. The interactive effects between meteorological and other factors on dengue incidence are similar with that of the population density. For example, the areas with high road density, GDP, urbanization, and urban village ratio were also usually located in the central urban areas with high population density [64]. These findings indicate that the combined effects of various factors should be considered in the risk assessment and control prevention strategies of dengue fever in South China.

This study has several strengths. First, we collected data at a fine spatial scale using the GIS and RS techniques and examined both independent and joint impacts of socio-ecological factors on dengue fever incidence, which extends our understanding of dengue transmission in South China. Second, to the best of our knowledge, this is the first study to investigate the effects of urban village, a special geographical phenomenon in the process of urbanization in China, on dengue transmission in China.

However, several limitations should be mentioned while interpreting our findings. First, adult mosquito data in this study were inadequate to further verify the effects of socio-ecological factors on dengue fever transmission because the establishment of vector surveillance in Guangdong was finished in 2015. Moreover, the mosquito population data were spatially uneven in Guangzhou area. However, we used BI to indirectly explain their mediation effects during the effects of socio-ecological factors on dengue fever transmission. Second, mosquito survival and dengue fever transmission has been reported to decrease when daily temperature is either cooler than 18 °C or warmer than 32 °C [65]. Therefore, the variation of temperature is another important factor affecting the behavior of mosquitos. In this study, we only used the daily mean temperature to explore its effects on dengue incidence rate. Therefore, more studies are needed to assess the effects of temperature variation, such as daily maximum and minimum temperature, in Guangzhou area.

## 5. Conclusions

Four clusters of dengue cases were identified in Guangzhou in 2014. The dengue incidence might either be independently or be jointly impacted by multiple socio-ecological factors, including meteorological, environmental, and socioeconomic factors. These findings extend our understanding of the nature of dengue fever transmission, and also have important implications for the planning and implementation of control and prevention measures in South China.

## Figures and Tables

**Table 1:** Spatial association between socio-ecological factors and dengue fever incidence.

Factors	GDP	Population Density	Precipitation	Road Density	Temperature	Urbanization Level	Vegetation Fraction	Urban Village Ratio	Water Body Areas
*R*	−0.23 **	0.49 **	0.09 **	0.36 **	0.51 **	0.42 **	−0.57 **	0.28 **	−0.38 **
*q*	0.03	0.03	0.24 *	0.24 *	0.33 *	0.11 *	0.19 *	0.13 *	0.23 *

*R* means correlation coefficient, *q* is the result calculated using the geographical detector, ** *p* < 0.01, * *p* < 0.05.

## Supplementary Materials

The following are available online at www.mdpi.com/1660-4601/14/7/795/s1, Table S1: Number of indigenous cases by gender, age and employment status in Guangzhou 2014. Table S2: Interactive impact of socio-ecological factors on dengue fever incidence.

## References

[1] Hopp M.J., Foley J.A. (2001). Global-scale relationships between climate and the dengue fever vector, *Aedes aegypti*. Clim. Chang..

[2] World Health Organization (WHO) (2012). A Who Report on Global Strategy for Dengue Prevention and Control, 2012–2020.

[3] Hales S., De W.N., Maindonald J., Woodward A. (2002). Potential effect of population and climate changes on global distribution of dengue fever: An empirical model. Lancet.

[4] Wu P.C., Lay J.G., Guo H.R., Lin C.Y., Lung S.C., Su H.J. (2009). Higher temperature and urbanization affect the spatial patterns of dengue fever transmission in subtropical Taiwan. Sci. Total Environ..

[5] Teurlai M., Menkès C.E., Cavarero V., Degallier N., Descloux E., Grangeon J.P., Guillaumot L., Libourel T., Lucio P.S., Mathieudaudé F. (2015). Socio-economic and climate factors associated with dengue fever spatial heterogeneity: A worked example in new caledonia. PLoS Negl. Trop. Dis..

[6] Jayakumary M., Jayadevan S. (2005). Role of the environment in a dengue fever epidemic. Epidemiology.

[7] Xiong Y., Chen Q. (2014). Epidemiology of dengue fever in China since 1978. J. South Med. Univ..

[8] Hunter J.M., Rey L., Scott D. (1982). Man-made lakes and man-made diseases. Towards a policy resolution. Soc. Sci. Med..

[9] Chandren J.R., Li P.W., Abubakar S. (2015). Practices of dengue fever prevention and the associated factors among the orang asli in Peninsular Malaysia. PLoS Negl. Trop. Dis..

[10] Choi Y., Tang C.S., Mciver L., Hashizume M., Chan V., Abeyasinghe R.R., Iddings S., Huy R. (2016). Effects of weather factors on dengue fever incidence and implications for interventions in Cambodia. BMC Public Health.

[11] Phung D., Guo Y., Nguyen H.T.L., Rutherford S., Baum S., Chu C. (2016). High temperature and risk of hospitalizations, and effect modifying potential of socio-economic conditions: A multi-province study in the tropical mekong delta region. Environ. Int..

[12] Fuller D.O., Troyo A., Beier J.C. (2009). El niño southern oscillation and vegetation dynamics as predictors of dengue fever cases in Costa Rica. Environ. Res. Lett..

[13] Qi X., Wang Y., Li Y., Meng Y., Chen Q., Ma J., Gao G.F. (2015). The effects of socioeconomic and environmental factors on the incidence of dengue fever in the Pearl River Delta, China, 2013. PLoS Negl. Trop. Dis..

[14] Hu W., Clements A., Williams G., Tong S., Mengersen K. (2012). Spatial patterns and socioecological drivers of dengue fever transmission in Queensland, Australia. Environ. Health Perspect..

[15] Lu L., Lin H., Tian L., Yang W., Sun J., Liu Q. (2009). Time series analysis of dengue fever and weather in Guangzhou, China. BMC Public Health.

[16] Jones B., O’Neill B.C. (2016). Spatially explicit global population scenarios consistent with the shared socioeconomic pathways. Environ. Res. Lett..

[17] Khormi H.M., Kumar L. (2011). Modeling dengue fever risk based on socioeconomic parameters, nationality and age groups: GIS and remote sensing based case study. Sci. Total Environ..

[18] Mondini A., Chiaravalloti F. (2008). Spatial correlation of incidence of dengue with socioeconomic, demographic and environmental variables in a Brazilian city. Sci. Total Environ..

[19] Pham H.V., Doan H.T., Phan T.T., Minh N.N. (2011). Ecological factors associated with dengue fever in a central highlands province, Vietnam. BMC Infect. Dis..

[20] Russell R.C., Webb C.E., Williams C.R., Ritchie S.A. (2005). Mark-release-recapture study to measure dispersal of the mosquito Aedes Aegypti in cairns, Queensland, Australia. Med. Vet. Entomol..

[21] Thammapalo S., Chongsuvivatwong V., Geater A., Dueravee M. (2008). Environmental factors and incidence of dengue fever and dengue haemorrhagic fever in an urban area, Southern Thailand. Epidemiol. Infect..

[22] Wang J.F., Li X.H., Christakos G., Liao Y.L., Zhang T., Gu X., Zheng X.Y. (2010). Geographical detectors-based health risk assessment and its application in the neural tube defects study of the Heshun region, China. Int. J. Geogr. Inf. Sci..

[23] Fan J., Lin H., Wang C., Bai L., Yang S., Chu C., Yang W., Liu Q. (2014). Identifying the high-risk areas and associated meteorological factors of dengue transmission in guangdong province, china from 2005 to 2011. Epidemiol. Infect..

[24] Waterman S.H., Gubler D.J. (1989). Dengue fever. Clin. Dermatol..

[25] Xiao J.P., He J.F., Deng A.P., Lin H.L., Song T., Peng Z.Q., Wu X.C., Liu T., Li Z.H., Rutherford S. (2016). Characterizing a large outbreak of dengue fever in Guangdong province, China. Infect. Dis. Poverty.

[26] Wang L., Wang Y., Jin S., Wu Z., Chin D.P., Koplan J.P., Wilson M.E. (2008). Emergence and control of infectious diseases in China. Lancet.

[27] Sun J., Lin J., Yan J., Fan W., Liang L., Lv H., Hou J., Feng L., Tao F., Chen Z. (2011). Dengue virus serotype 3 subtype iii, Zhejiang province, China. Emerg. Infect. Dis..

[28] Lai S., Huang Z., Hang Z., Anders K.L., Perkins T.A., Yin W., Yu L., Di M., Chen Q., Zhang Z. (2015). The changing epidemiology of dengue in China, 1990–2014: A descriptive analysis of 25 years of nationwide surveillance data. BMC Med..

[29] Marcantonio M. (2016). First assessment of potential distribution and dispersal capacity of the emerging invasive mosquito aedes koreicus in Northeast Italy. Parasit. Vectors.

[30] Wickham J.D., Stehman S.V., Gass L., Dewitz J., Fry J.A., Wade T.G. (2013). Accuracy assessment of NLCD 2006 land cover and impervious surface. Remote Sens. Environ..

[31] Song Y., Zenou Y., Ding C. (2008). Let’s not throw the baby out with the bath water: The role of urban villages in housing rural migrants in China. Urban Stud..

[32] Jiang Z., Huete A.R., Chen J., Chen Y., Li J., Yan G., Zhang X. (2006). Analysis of NDVI and scaled difference vegetation index retrievals of vegetation fraction. Remote Sens. Environ..

[33] Banu S., Hu W., Hurst C., Tong S. (2011). Dengue transmission in the Asia-Pacific region: Impact of climate change and socio-environmental factors. Trop. Med. Int. Health.

[34] He H., Guo Z., Xiao W., Guo Q. (2005). Mapping monthly precipitaion for Tibetan plateay with GIS and multivariate analysis based on dem data. Acta Ecol. Sin..

[35] Anselin L. (1995). Local indicators of spatial association—Lisa. Geogr. Anal..

[36] Wang J.F., Yan W., Jing Z., Christakos G., Sun J.L., Xin L., Lin L., Fu X.Q., Shi Y.Q., Li X.M. (2013). Correction: Spatiotemporal transmission and determinants of typhoid and paratyphoid fever in Hongta district, Yunnan province, China. PLoS Negl. Trop. Dis..

[37] Huang J., Wang J., Bo Y., Xu C., Hu M., Huang D. (2014). Identification of health risks of hand, foot and mouth disease in China using the geographical detector technique. Int. J. Environ. Res. Public Health.

[38] Liao Y., Yan Z., Lei H., Wang J., Xin L., Zhang N., Bing X. (2016). Temporal and spatial analysis of neural tube defects and detection of geographical factors in Shanxi province, China. PLoS ONE.

[39] Da Costa A.I., Natal D. (1998). Geographical distribution of dengue and socioeconomic factors in an urban locality in Southeastern Brazil. Revista De Saúde Pública.

[40] Zellweger R.M., Cano J., Mangeas M., Taglioni F., Mercier A., Despinoy M., Menkès C.E., Dupontrouzeyrol M., Nikolay B., Teurlai M. (2017). Socioeconomic and environmental determinants of dengue transmission in an urban setting: An ecological study in Nouméa, New Caledonia. PLoS Negl. Trop. Dis..

[41] Lin H., Liu T., Song T., Lin L., Xiao J., Lin J., He J., Zhong H., Hu W., Deng A. (2016). Community involvement in dengue outbreak control: An integrated rigorous intervention strategy. PLoS Negl. Trop. Dis..

[42] Sarfraz M.S. (2013). Near real-time characterisation of urban environments: A holistic approach for monitoring dengue fever risk areas. Int. J. Digit. Earth.

[43] Hu W., Clements A., Williams G., Tong S. (2010). Dengue fever and el nino/southern oscillation in Queensland, Australia: A time series predictive model. Occup. Environ. Med..

[44] Chung H. (2009). The planning of “villages-in-the-city” in Shenzhen, China: The significance of the new state-led approach. Int. Plan. Stud..

[45] Zheng N., Lin Y., Luo B. (2003). The ecological study on the mosquito species of dengue fever in Fuzhou. Strait. J. Prev. Med..

[46] Gong D., Zhou H. (2009). Progress in dengue fever important vector *Aedes albopictus* in China. Chin. J. Vector Biol. Control.

[47] Velascosalas Z.I., Sierra G.M., Guzmán D.M., Zambrano J., Vivas D., Comach G., Wilschut J.C., Tami A. (2014). Dengue seroprevalence and risk factors for past and recent viral transmission in Venezuela: A comprehensive community-based study. Am. J. Trop. Med. Hyg..

[48] Liu T., Xu Y.J., Zhang Y.H., Yan Q.H., Song X.L., Xie H.Y., Luo Y., Rutherford S., Chu C., Lin H.L. (2013). Associations between risk perception, spontaneous adaptation behavior to heat waves and heatstroke in Guangdong province, China. BMC Public Health.

[49] Troyo A., Fuller D.O., Calderónarguedas O., Solano M.E., Beier J.C. (2009). Urban structure and dengue fever in puntarenas, costa rica. Singap. J. Trop. Geogr..

[50] Reiter P., Gubler D.J., Ooi E.E., Vasudevan S., Farrar J. (1997). Surveillance and control of urban dengue vectors. JAMA Intern. Med..

[51] Muir L.E., Kay B.H. (1998). Aedes aegypti survival and dispersal estimated by mark-release-recapture in Northern Australia. Am. J. Trop. Med. Hyg..

[52] Harrington L.C., Buonaccorsi J.P., Edman J.D., Costero A., Kittayapong P., Clark G.G., Scott T.W. (2001). Analysis of survival of young and old *Aedes aegypti* (diptera: Culicidae) from Puerto Rico and Thailand. J. Med. Entomol..

[53] Tian H., Huang S., Zhou S., Bi P., Yang Z., Li X., Chen L., Cazelles B., Yang J., Luo L. (2016). Surface water areas significantly impacted 2014 dengue outbreaks in Guangzhou, China. Environ. Res..

[54] Shen J.C., Luo L., Li L., Jing Q.L., Ou C.Q., Yang Z.C., Chen X.G. (2015). The impacts of mosquito density and meteorological factors on dengue fever epidemics in Guangzhou, China, 2006–2014: A time-series analysis. BioMed Environ. Sci..

[55] Focks D.A., Daniels E., Haile D.G., Keesling J.E. (1995). A simulation model of the epidemiology of urban dengue fever: Literature analysis, model development, preliminary validation, and samples of simulation results. Am. J. Trop. Med. Hyg..

[56] Nakhapakorn K., Tripathi N.K. (2005). An information value based analysis of physical and climatic factors affecting dengue fever and dengue haemorrhagic fever incidence. Int. J. Health Geogr..

[57] Xu L., Stige L.C., Chan K.S., Zhou J., Yang J., Sang S., Wang M., Yang Z., Yan Z., Jiang T. (2017). Climate variation drives dengue dynamics. Proc. Natl. Acad. Sci. USA.

[58] Wu P.C., Guo H.R., Lung S.C., Lin C.Y., Su H.J. (2007). Weather as an effective predictor for occurrence of dengue fever in Taiwan. Acta Trop..

[59] Lowe R., Bailey T.C., Stephenson D.B., Graham R.J., Coelho C.A.S., Carvalho M., Barcellos C. (2011). Spatio-temporal modelling of climate-sensitive disease risk: Towards an early warning system for dengue in Brazil. Comput. Geosci..

[60] Shaman J., Day J.F. (2007). Reproductive phase locking of mosquito populations in response to rainfall frequency. PLoS ONE.

[61] Sang S., Gu S., Peng B., Yang W., Yang Z., Lei X., Yang J., Liu X., Tong J., Wu H. (2015). Predicting unprecedented dengue outbreak using imported cases and climatic factors in Guangzhou, 2014. PLoS Negl. Trop. Dis..

[62] Machado-Machado E.A. (2011). Empirical mapping of suitability to dengue fever in Mexico using species distribution modeling. Appl. Geogr..

[63] Depradine C., Lovell E. (2004). Climatological variables and the incidence of dengue fever in Barbados. Int. J. Environ. Health Res..

[64] Ji W., Wang Y., Zhuang D., Song D., Shen X., Wang W., Li G. (2014). Spatial and temporal distribution of expressway and its relationships to land cover and population: A case study of Beijing, China. Transp. Res. Part D.

[65] Eastin M.D., Delmelle E., Casas I., Wexler J., Self C. (2014). Intra- and interseasonal autoregressive prediction of dengue outbreaks using local weather and regional climate for a tropical environment in Colombia. Am. J. Trop. Med. Hyg..

