# Supplementary material for: Individual and Interactive Effects of Socio-Ecological Factors on Dengue Fever at Fine Spatial Scale: A Geographical Detector-Based Analysis
Source: Int J Environ Res Public Health. 2017 Jul 17;14(7):795. doi: 10.3390/ijerph14070795 (PMC5551233; doi:10.3390/ijerph14070795)
Supplement: Supplementary file 1 [file ijerph-14-00795-s001.pdf]

Table S1 Number of indigenous cases by gender, age and employment status in Guangzhou 2014

Variables	Indigenous cases (incidence rate/10^5)
Total cases	37322 (289.97）
Gender	
Male	18388 (273.98)
Female	18934 (307.39)
Age group (years)	
<15	2693 (189.86)
15-44	20024 (255.97)
45-64	10111 (370.79)
>64	4494 (497.64)
Employment status (constituent ratio %)	
Household/unemployed	8717 (23.36)
Retired	5221 (13.99)
Business service	4436 (11.89)
Worker	4194 (11.24)
Student	2788 (7.47)
Farmer	1356 (3.63)）
Administrative staff	1762 (4.72)
Other	1419 (3.80)
Unknown	7429 (19.90)

Table S2. Interactive impact of socio-ecological factors on dengue fever incidence

$X_1 \cap X_2 = X_3$	X_1	X_2	Relation among X_1, X_2, X_3
GDP ∩ Urb=0.15**	0.03	0.11	$X_3 > X_1 + X_2$ Enhance, nonlinear
GDP ∩ Uv=0.15	0.03	0.13	$X_3 > Max(X_1, X_2)$ Enhance, bi
GDP ∩ Roa=0.29**	0.03	0.24	$X_3 > X_1 + X_2$ Enhance, nonlinear
GDP ∩ Pop=0.07	0.03	0.03	$X_3 > X_1 + X_2$ Enhance, nonlinear
GDP ∩ Water=0.25**	0.03	0.23	$X_3 > Max(X_1, X_2)$ Enhance, bi
GDP ∩ Veg=0.24**	0.03	0.19	$X_3 > X_1 + X_2$ Enhance, nonlinear
GDP ∩ Temp=0.35**	0.03	0.33	$X_3 > Max(X_1, X_2)$ Enhance, bi
GDP ∩ Preci 0.26**	0.03	0.24	$X_3 > Max(X_1, X_2)$ Enhance, bi
Urb ∩ Uv=0.19**	0.11	0.13	$X_3 > Max(X_1, X_2)$ Enhance, bi
Urb ∩ Roa=0.31**	0.11	0.24	$X_3 > Max(X_1, X_2)$ Enhance, bi
Urb ∩ Pop=0.13**	0.11	0.03	$X_3 > Max(X_1, X_2)$ Enhance, bi
Urb ∩ Water=0.25**	0.11	0.23	$X_3 > Max(X_1, X_2)$ Enhance, bi
Urb ∩ Veg=0.29	0.11	0.19	$X_3 > Max(X_1, X_2)$ Enhance, bi
Urb ∩ Temp=0.35**	0.11	0.33	$X_3 > Max(X_1, X_2)$ Enhance, bi
Urb ∩ Preci=0.31**	0.11	0.24	$X_3 > Max(X_1, X_2)$ Enhance, bi
Uv ∩ Roa=0.30**	0.13	0.24	$X_3 > Max(X_1, X_2)$ Enhance, bi
Uv ∩ Pop=0.14**	0.13	0.03	$X_3 > Max(X_1, X_2)$ Enhance, bi
Uv ∩ Water=0.28**	0.13	0.23	$X_3 > Max(X_1, X_2)$ Enhance, bi
Uv ∩ Veg=0.24*	0.13	0.19	$X_3 > Max(X_1, X_2)$ Enhance, bi
Uv ∩ Temp=0.38**	0.13	0.33	$X_3 > Max(X_1, X_2)$ Enhance, bi
Uv ∩ Preci=0.31**	0.13	0.24	$X_3 > Max(X_1, X_2)$ Enhance, bi
Roa ∩ Pop=0.25**	0.24	0.03	$X_3 > Max(X_1, X_2)$ Enhance, bi
Roa ∩ Water=0.35**	0.24	0.23	$X_3 > Max(X_1, X_2)$ Enhance, bi
Roa ∩ Veg=0.33	0.24	0.19	$X_3 > Max(X_1, X_2)$ Enhance, bi
Roa ∩ Temp=0.41**	0.24	0.33	$X_3 > Max(X_1, X_2)$ Enhance, bi
Roa ∩ Preci=0.40**	0.24	0.24	$X_3 > Max(X_1, X_2)$ Enhance, bi
Pop ∩ Water=0.24**	0.03	0.23	$X_3 > Max(X_1, X_2)$ Enhance, bi

Pop ∩ Veg=0.21**	0.03	0.19	X_3>Max(X_1, X_2) Enhance, bi
Pop ∩ Temp=0.34**	0.03	0.33	X_3>Max(X_1, X_2) Enhance, bi
Pop ∩ Preci=0.28**	0.03	0.24	X_3> X_1+ X_2 Enhance, nonlinear
Water ∩ Veg=0.29**	0.23	0.19	X_3>Max(X_1, X_2) Enhance, bi
Water ∩ Temp=0.39**	0.23	0.33	X_3>Max(X_1, X_2) Enhance, bi
Water ∩ Preci=0.40**	0.23	0.24	X_3>Max(X_1, X_2) Enhance, bi
Veg ∩ Temp=0.34**	0.19	0.33	X_3>Max(X_1, X_2) Enhance, bi
Veg ∩ Preci=0.33**	0.19	0.24	X_3>Max(X_1, X_2) Enhance, bi
Temp ∩ Preci=0.42**	0.33	0.24	X_3>Max(X_1, X_2) Enhance, bi

**means P<0.01, * means P<0.05, GDP means GDP per capita, Urb means urbanization level, Uv means urban village ratio, Roa means road density, Pop means population density, Water means water body area, Veg means vegetation fraction, Temp means temperature, Preci means precipitation.
